# Detection and Identification of Diverse Phytoplasmas in Declining Persimmon Plants

**DOI:** 10.3390/microorganisms13030645

**Published:** 2025-03-12

**Authors:** Seyyed Alireza Esmaeilzadeh-Hosseini, Ghobad Babaei, Sri Tej Mateeti, Francesco Pacini, Assunta Bertaccini

**Affiliations:** 1 Plant Protection Research Department, Yazd Agricultural and Natural Resources Research and Education Centre, Agricultural Research, Education and Extension Organization (AREEO), Yazd 8915813156, Iran; 2 Plant Protection Research Department, Chaharmahal and Bakhtiari Agricultural and Natural Resources Research and Education Centre, Agricultural Research, Education and Extension Organization (AREEO), Shahrekord 8813657351, Iran; ghobad.babaee@gmail.com; 3 Department of Agricultural and Food Sciences, *Alma Mater Studiorum*—University of Bologna, 40127 Bologna, Italy; sritej.mateeti2@unibo.it (S.T.M.); francesco.pacini2@unibo.it (F.P.)

**Keywords:** 16SrXXIX group, 16SrII-D subgroup, *Diospyros kaki*, multiple infection, Yazd, Iran

## Abstract

Persimmon (*Diospyros kaki*) plants showing yellowing, reddening, die-back, and decline symptoms were observed in Mehriz (Yazd province), Iran. Total DNAs, extracted from samples collected from symptomatic and symptomless plants, were subjected to direct and nested PCR, amplifying the 16S rRNA gene of phytoplasmas using specific primer pairs. PCR amplicons of expected lengths were obtained, mainly from nested PCR, and only from samples collected from symptomatic plants. Real and virtual RFLP, phylogenetic, and DNA identity analyses of the partial 16S rRNA gene sequences suggested the presence of diverse phytoplasmas in the analyzed samples. The identified phytoplasmas were referable to ‘*Candidatus* Phytoplasma omanense’ (16SrXXIX group) and ‘*Ca*. P. australasiae = australasiaticum’ (16SrII-D subgroup). The results of the sampling and testing highlight the urgent need for an accurate survey to verify the presence and identity of phytoplasmas in symptomatic fruit trees in Iran, in order to be able to plan appropriate management strategies. Further investigations of the possible role of ‘*Ca*. P. omanense’ strains as an emerging threat to fruit orchards in Iran should also be performed.

## 1. Introduction

The persimmon is an edible fruit in the genus *Diospyros*, and the most widely cultivated species is the kaki persimmon (*Diospyros kaki*, Ebenaceae). The persimmon tree height reaches 4.5 to 18 m, and the leaves are 7–15 cm long, oblong in shape with 2 cm brown hairy petioles [1]. The cultivated persimmon area in Iran is 2311 hectares, and the amount of production is 37,150 tons [2]. Among the several factors causing severe damage to plants, phytoplasma diseases are showing increasing relevance especially in perennial agricultural woody species [3]. Phytoplasmas are cell wall-less plant pathogenic bacteria that inhabit the phloem tissue of infected plants and are transmitted mainly by leafhoppers, planthoppers and psyllids. It has been shown that phytoplasmas can also be spread by seeds, vegetative propagation, grafting, cuttings, and via plant parasitic species [4,5,6,7,8]. Phytoplasma diseases have different geographic distributions, and the associated bacteria are classified into the provisional genus ‘*Candidatus* Phytoplasma’ by comparison of their 16S rRNA gene sequences [9,10]. Since the first observation of phytoplasmas [11] and their associated diseases in sesame in Iran [12], many phytoplasma-associated diseases have been identified in various plant species in the country. Some of these phytoplasma diseases are very destructive and have great economic importance. So far, several strains of ‘*Ca*. P. asteris’, ‘*Ca*. P. australasiae = australasiaticum’, ‘*Ca*. P. pruni’, ‘*Ca*. P. trifolii’, ‘*Ca*. P. fraxini’, ‘*Ca*. P. phoenicium’, ‘*Ca*. P. prunorum’, ‘*Ca*. P. oryzae’, ‘*Ca*. P. solani’, ‘*Ca*. P. cynodontis’, ‘*Ca*. P. omanense’ and ‘*Ca*. P. tamaricis’, have been identified across at least 44 plant families in Iran. The most widespread and associated with relevant crops are ‘*Ca*. P. asteris’, ‘*Ca*. P. australasiae = australasiaticum’ and ‘*Ca*. P. solani’, detected in both herbaceous and woody host species of agronomical importance [13,14,15]. There is not much information about the presence and infection of persimmon trees with phytoplasmas. However, persimmon plants with stem fasciation, internodes shortening, and little leaves were shown to be infected in China with a ’*Ca*. P. ziziphi’ strain [16,17]. In Jordan, molecular analyses identified the presence of ‘*Ca*. P. solani’ in persimmon trees showing leaf scorch and rolling [18]. In Iran, previous research has documented the presence of ‘*Ca*. P. omanense’ in persimmon orchards in the Yazd province [19]. However, the phytoplasma diversity affecting kaki in this province and in the country remains understudied. This study aims to verify the presence and identity of phytoplasmas in a number of persimmon orchards in which there were high percentages of plants showing yellowing, reddening, and die-back. The results of a field survey followed by molecular analyses in Mehriz (Yazd province), Iran are reported here.

## 2. Materials and Methods

### 2.1. Material Sampling and Phytoplasma Control Strains

Persimmon plant samples were collected by randomly sampling across five 1000 m^2^ fruit orchards in which several plants showed decline symptoms. The fruit tree species in the orchard were mixed and included peach, plum, apple, pear, pomegranate and apricot. Five locations were selected for the sampling, and in each of them, 1000 m of each orchard were selected randomly by sampling following a diagonal transect. A total of five to seven leaves were removed from the branches of each of five symptomatic trees every 1000 m randomly and transferred to the laboratory for molecular testing. One asymptomatic persimmon tree was also sampled every 1000 m. The GPS coordinates of the five orchards all located in Mehriz (Yard province) are 31°33′27.3″ N 54°26′27.4″ E; 31°33′31.1″ N 54°26′33.7″ E; 31°33′30.2″ N 54°26′31.6″ E; 31°33′28.4″ N 54°26′28.6″ E and 31°33′33.0″ N 54°26′27.0″ E. A total of 25 symptomatic and five symptomless plants were sampled. The number of symptomatic persimmon plants observed over the total number of persimmon plants was verified to calculate the percentage of symptomatic trees. As positive and reference controls, the following phytoplasmas enclosed in the EPPO-Qbank collection [20,21] were used: ‘*Ca*. P. omanense’ strain IM-1 (16SrXXIX-A), ‘*Ca*. P. asteris’ strain AAY (16SrI-B), ‘*Ca*. P. tritici’ strain KVM (16SrI-C), ‘*Ca*. P. aurantifolia = citri’ strain WBDL (16SrII-B), ‘*Ca*. P. australasiae = australsiaticum’ strain TBB (16SrII-D), *Pichris echoides* phyllody (PEP) 16SrII-E, and ‘*Ca*. P. solani’ strain STOL (16SrXII-A).

### 2.2. Nucleic Acid Extraction and Molecular Analyses

Total DNA was extracted from 0.2 g midrib tissue from symptomatic and asymptomatic plants using the procedure described by Zhang et al. [22]. These samples were tested for the presence of phytoplasmas by PCR using the primer pairs P1/P7 [23,24] followed by nested PCR with primer pairs R16mF2/R16mR2 and R16F2n/R16R2 [25]. The primer pair P1/P7 amplifies an 1800 bp fragment of the ribosomal operon, which includes the 16S rRNA gene, the 16S-23S intergenic spacer region, and a portion of the 5′ regions of the 23S rRNA gene. R16mF2/R16mR2 and R16F2n/R16R2 primer pairs amplify about 1400 and 1250 bp of 16S rRNA gene, respectively. PCR mixes for all the primers employed were as previously described [26]; PCR conditions were set using the following parameters: 1 min at 94 °C (2 min of initial denaturation), 2 min at 55 °C and 3 min at 72 °C (10 min of final extension). PCR conditions for the nested PCR were set with the annealing temperature raised to 58 °C. The samples in which phytoplasmas were identified on the 16S rRNA gene were employed for nested PCR amplification of the *tuf* gene using the primer cocktails Tuf340/Tuf890 in PCR and Tuf400/Tuf835 for the nested reaction [27] and *secA* gene using SecAfor1/SecA rev3 and SecAfor5/SecArev2 [28,29] primers under reported conditions [27,28]. Asymptomatic persimmon samples and H_2_O were employed in all reactions as negative controls. The amplifications were carried out in a thermal cycler (Bio-Rad, Hercules CA, USA) and the PCR products were electrophoresed in 1% agarose gels in TBE buffer and visualized with a UV transilluminator following ethidium bromide staining. The R16F2n/R16R2 amplified products were digested separately with each of the *Tru1*I, *Hha*I, *Rsa*I, and *Taq*I restriction enzymes, and the *tuf* gene amplicons were digested with *Tru1*I and *Tsp509*I restriction enzymes (Fermentas, Vilnius, Lithuania). The restriction products were then separated by 6.7% polyacrylamide gel electrophoresis and stained and visualized as described above. The resulting RFLP patterns were compared with those previously published from other phytoplasmas and with reference phytoplasma strains from EPPO-QBank [20,21,30].

### 2.3. Phylogenetic Analyses and Virtual Restriction Fragment Length Polymorphism Analyses

After RFLP analyses, the nested PCR amplicons of six samples from Mehriz fields were directly sequenced using the same R16mF2/R16mR2 primer pair used for their nested amplification. After alignment and assembling, three of these sequences, shown to be of higher quality, were used for phylogenetic analyses with MEGA software version 7.0 [31]. *Acholeplasma laidlawii* was used as an outgroup to root the trees. A database search of homologous sequences was performed by web Blastn (https://blast.ncbi.nlm.nih.gov/Blast.cgi, accessed on 28 December 2024) analyses at the National Centre for Biotechnology Information (NCBI). The phylogenetic trees were constructed by the Neighbor-Joining method. Bootstrapping was performed 1000 times to estimate clade stability and support for the branches. A further evolutionary history of available strains of ‘*Ca*. P. omanense’ was inferred using the Maximum Likelihood method [32] of the same program. Initial tree(s) for the heuristic search were obtained automatically by applying Neighbor-Join and BioNJ algorithms to a matrix of pairwise distances estimated using the Maximum Composite Likelihood (MCL) approach and then selecting the topology with superior log likelihood value.

Virtual RFLP analysis with the *i*PhyClassifier [33] was used to determine the subgroup affiliation of sequenced phytoplasmas. Each aligned DNA fragment was digested *in silico* with 17 distinct restriction enzymes: *Alu*I, *BamH*I, *Bfa*I, *BstU*I (*Tha*I), *Dra*I, *EcoR*I, *Hae*III, *Hha*I, *Hinf*I, *Hpa*I, *Hpa*II, *Kpn*I, *Mbo*I (*Sau3A*I), *Mse*I (*Tru1*I), *Rsa*I, *Ssp*I and *Taq*I. To further verify the presence of differences in the amplified 16S rDNA, the sequences obtained were compared with the reference strain of ‘*Ca*. P. omanense’; 16SrXXIX-A (GenBank accession number EF666051) and the other strains of the same phytoplasma available in GenBank (Table 1) with further enzymes resulted to be informative for this ‘*Ca*. Phytoplasma’ [34] in virtual RFLP using the program pDRAW32 (http://www.acaclone.com/).

## 3. Results

**Figure 1:**
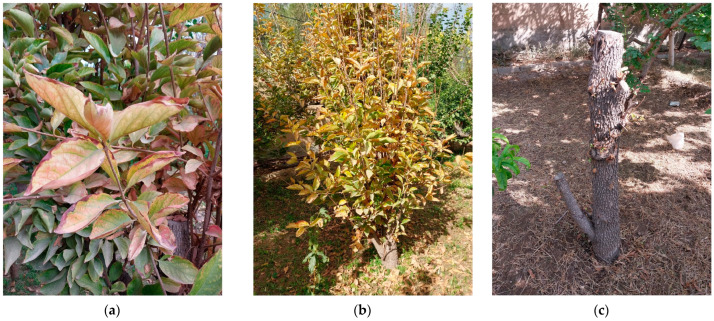
Persimmon (*Diospyros kaki*) plants showing yellowing and reddening (**a**), die-back (**b**), and full decline symptoms (**c**) in Mehriz (Yazd province), Iran.

Persimmon plants showing yellowing, reddening, die-back, and decline symptoms (Figure 1) were observed up to 21% in Mehriz orchards (Yazd province, Iran). The molecular analyses reveal the presence of phytoplasmas in all the 25 symptomatic persimmon plants sampled, while the asymptomatic samples and H_2_O yielded negative results. These results allow to confirm the association of phytoplasma presence with the observed decline symptomatology.

**Figure 2:**
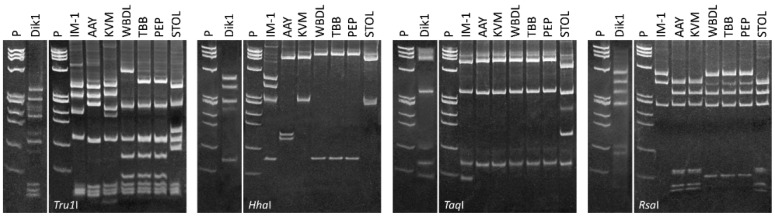
RFLP profiles visualized in polyacrylamide gels of R16F2n/R2 amplicons of the phytoplasmas detected in persimmon sample Dik1 using the restriction enzymes listed below the gels in comparison with the reference strains listed in materials and methods. P, marker phiX174 DNA *Hae*III-digested, with fragment sizes, from top to bottom, of 1353, 1078, 872, 603, 310, 281, 271, 234, 194, 118 and 72 bp.

DNA fragments of the expected length were amplified from some of the symptomatic persimmon plants in PCR using P1/P7 and from all of them after nested PCR with R16mF2/R16mR2 and R16F2n/R16R2, respectively. The R16F2n/R16R2 nested PCR products from persimmon phytoplasmas were analyzed by RFLP with the four restriction enzymes listed above. The majority of the RFLP patterns were shown to be identical to each other, with a number of bands that indicate the presence of mixed phytoplasma infection. However, in some cases, it was possible to distinguish a profile with stronger and more visible bands together with a second profile suggesting the presence of two diverse phytoplasmas. It was therefore possible to verify that the main recurrent strongest profile was referable to the one of phytoplasmas in group 16SrXXIX (identical to the reference strain IM-1, 16SrXXIX-A). The second profile present in a number of samples with lighter banding was also referable in some of the enzymes used such as *Tru1*I and *Hha*I, to the profile of ‘*Ca*. P. solani’ (strain STOL, 16SrXI-A), confirming the presence of a possible mixed infection of the two phytoplasmas (Figure 2). A further profile was observed in some of the samples, which was referable to phytoplasmas enclosed in group 16SrII.

The results of PCR via nested amplification for the *secA* gene were negative, while the *tuf* gene’s amplification produced expected fragments of 420–444 bp. The RFLP analyses of these latter amplicons with *Tru1*I and *Tsp509*I produced profiles not referable to single or mixed phytoplasma profiles, and the sequencing of these amplicons resulted in non-readable results.

Three among the R16mF2/R16mR2 amplified and sequenced fragments from phytoplasmas infecting persimmon showing the higher-quality sequences were submitted to GenBank under accession numbers (acc. nos.) PP829290, PP829288, and PP829289. A BLAST search using the PP829290 sequence showed its 99.92% identity with the ‘*Ca*. P. omanense’ strain Ft4 (*Prunus persica* yellowing and decline phytoplasma) (S. A. Esmailzadeh Hosseini, unpublished), and 99.52% with ‘*Ca*. P. omanense’ strain IM-1 (acc. no. EF666051), both members of the 16SrXXIX-A subgroup. The same phytoplasma strain also shows 99.60% identity to a strain from pomegranate from Jordan (acc. no. OL873126) and 99.76% to a bindweed strain (acc. no. KY047493) from Iran. A BLAST search using acc. no. PP829288 and acc. no. PP829289 sequences showed 100% identity with ‘*Ca*. P. australasiae = australasiaticum’ reference strain (acc. nos. Y10096-97).

**Figure 3:**
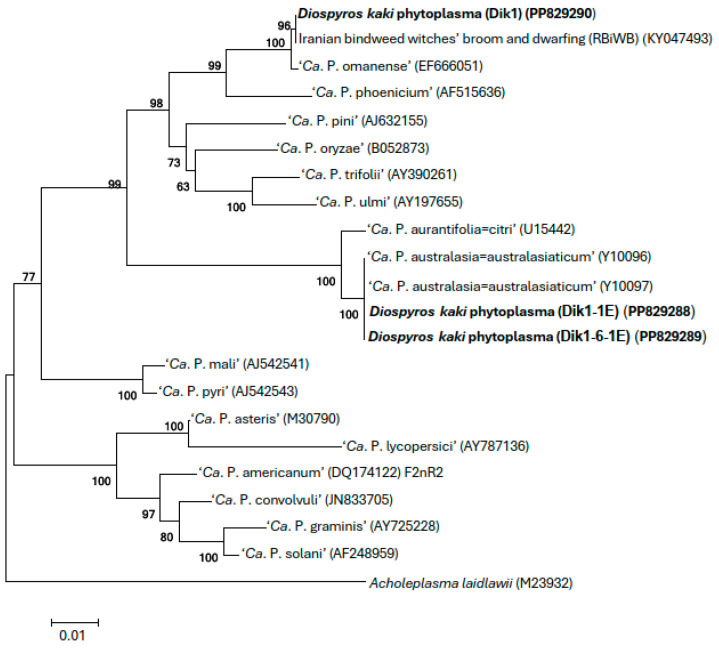
Phylogenetic trees constructed by the Neighbor-Joining method [32] using partial 16S rRNA gene sequences of several ‘*Candidatus* Phytoplasma’ species and *A. laidlaw*ii as outgroup. The percentages of replicate trees in which the associated taxa clustered together in the bootstrap test (1000 replicates) are shown next to the branches. The strains under study are in bold. GenBank accession numbers for sequences are given in parentheses on the right.

**Figure 4:**
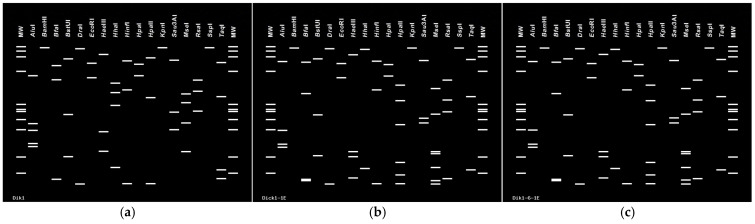
Virtual RFLP analyses on a 4% gel of R16F2n/R16R2 fragments of phytoplasmas from persimmon (*Diospyros kaki*) showing yellowing, reddening, die-back, and decline in Mehriz (Yazd province), Iran. (**a**) amplicon Dik1 pattern derived from the 16S rDNA F2n/R2 sequence with a similarity coefficient of 1.00 (identical) to the reference pattern of phytoplasmas enclosed in the 16SrXXIX-A subgroup (acc. no. EF666051); (**b**) amplicons Dik1-1E and (**c**) Dik1-6-1Epatterns derived from the 16S rDNA F2n/R2 sequences with similarity coefficients of 1.00 (identical) to the reference pattern of phytoplasmas in subgroup 16SrII-D (acc. no. Y10097). Enzymes used are listed at the top of each figure. MW, marker phiX174 DNA *Hae*III-digested, with fragment sizes, from top to bottom, of 1353, 1078, 872, 603, 310, 281, 271, 234, 194, 118 and 72 bp.

The phylogenetic trees generated using the 16S rDNA sequences of the phytoplasma strains detected in persimmon showed that these phytoplasma strains cluster to ‘*Ca*. P. omanense’ and ‘*Ca*. P. australasiae = australasiaticum’ (Figure 3), respectively. The virtual RFLP analyses of the 1.25 kbp 16S rDNA of the same sequences in the *i*PhyClassifier indicate that these phytoplasma strains belong to 16SrXXIX-A and 16SrII-D subgroups, respectively, confirming the results of RFLP analyses in the polyacrylamide gels (Figure 4).

**Figure 5:**
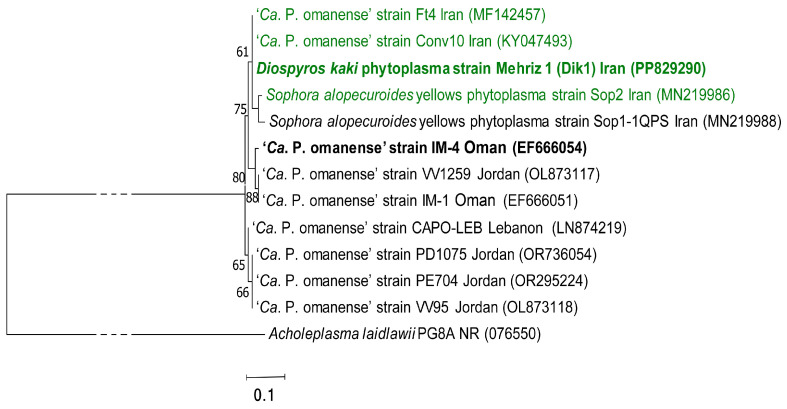
Molecular phylogenetic analysis by maximum likelihood method. The percentages of trees on which the associated taxa clustered together are shown next to the branches. Initial tree(s) for the heuristic search were obtained automatically by applying Neighbor-Join and BioNJ algorithms to a matrix of pairwise distances estimated using the Maximum Composite Likelihood (MCL) approach and then selecting the topology with superior log likelihood value. The tree is drawn to scale, with branch lengths measured in the number of substitutions per site. Evolutionary analyses were conducted in MEGA 7, and *A. laidlawii* is added as an outgroup to root the tree. GenBank accession numbers for sequences are given in parentheses on the right.

Moreover, comparing the sequences available in GenBank for all the strains of ‘*Ca*. P. omanense’ showing SNPs or GAPs with each others after alignment, it was possible to verify that diverse lineages of this phytoplasma were detected in different geographic areas of the Middle East. Moreover, they show a clear distribution in three clusters supported by a phylogenetic analyses bootstrap above 60 (Figure 5). The phytoplasma strains in these clusters are geographically distinct; ‘*Ca*. P. omanense’ strains from Iran are clustering together and are separated from the two strains from Oman and the one from Jordan (acc. nos. EF666051 and -54, and OL873117), while the remaining strains from Lebanon and Jordan cluster together.

The virtual RFLP analyses performed using the previously reported enzymes *Aat*II and *Bcg*I [34] in the pDraw program further confirm the presence of diverse genotypes (lineages) in the available sequences enclosed in the three clusters determined by phylogeny. The same 12 sequences trimmed at the same length as for the phylogenetic tree in Figure 5 show that the ‘*Ca*. P. omanense’ strains are further differentiable into six lineages (Table 1) showing an unexpected amount of variability in the 16S rRNA gene. In particular, the ‘*Ca*. P. omanense’ IM1 strain with acc. no. EF666051 (16SrXXIX-A), which is the reference strain, is differentiable from a second strain identified in the same plant species (*Cassia italica*) in Oman (acc. no. EF666054) by using these enzymes. On the other hand, this second strain described in Oman is identical to phytoplasma strains detected in various fruit trees form Jordan, recently reported as subgroup 16SrXXIX-B. Moreover, the strain identified in persimmon appears to be in the same lineage as strains from *Prunus persica*, *Vitis vinifera* and *Prunus dulcis* from Iran and Lebanon, respectively.

## 4. Discussion

The phytoplasmas in symptomatic persimmon plants were identified as ‘*Ca*. P. omanense’ (16SrXXIX-A subgroup) and ‘*Ca*. P. australasiae = australasiaticum’ (16SrII-D subgroup) by direct amplicon sequencing. They were identified from the sequences of the amplicons with higher quality and obtained via nested PCR from selected samples among the 25 positive samples. The majority of the samples showed RFLP profiles not clearly distinguishable as a single phytoplasma profile; in some cases, however, it was possible to distinguish the presence of profiles referable to 16SrII and 16SrXXIX phytoplasma ribosomal groups. Since there are no ribosomal primers specifically amplifying phytoplasmas in these ribosomal groups, it was not possible to verify the specific presence of each of the two phytoplasmas when they were amplified in a mixed infection on the 16S rRNA gene. The analyses performed on the *secA* gene were negative, while the *tuf* gene amplicons obtained were not suitable for the further verification of phytoplasma identity because of the lack of readable sequencing results, and the RFLP results were not clearly attributable to either of the two phytoplasma groups detected. These results indirectly confirm the presence of multiple microorganims in the samples tested, which made the phytoplasma identification quite difficult. The amplification with non-ribosomal genes is in several cases difficult to achieve, because in the phytoplasma genome they are present as a single copy; therefore, their sensitivity, and very likely also their specificity, are reduced. The detection and identification of phytoplasma strains in an accurate and sensitive manner are useful to study the epidemiological implications of phytoplasma diseases to design appropriate management. Detecting phytoplasma presence in tree host species, especially fruit and forest trees that have woody structures, is very often challenging due to their low concentration and their erratic distribution within infected plants [35]. The results obtained confirm the difficulties in detecting and identifying phytoplasmas when the plants are harboring diverse phytoplasma strains and/or bacterial strains present as possible endophytes.

However, both phytoplasmas identified are very important in Iran for different reasons. The ‘*Ca*. P. omanense’ includes phytoplasmas divided into three subgroups: 16SXXIX-A, identified in *Cassia* witches’ broom (CaWB) (acc. no. EF666051) in Oman and representing the reference strain [36]; 16SXXIX-B, Iranian bindweed witches’ broom and dwarfing (RBiWB) (acc. no. KY047493) detected in 2014 in Bafg (Yazd province, Iran) in alfalfa fields [14]; and 16SXXIX-B(C), almond witches’ broom yellowing and die-back (AL163) (acc. no. OL873126), recently detected in Jordan [18]. Moreover ‘*Ca*. P. omanense’ strains were also detected in grapevine with yellows, stunted bindweed, and Cixiidae planthoppers in Lebanon [37]. The importance of these phytoplasmas is related to the fact that they were first identified less than 20 years ago in the Arabian Peninsula, but now they show increased spread in several countries of the area enclosing Iran. The identified hosts and symptoms of ‘*Ca*. P. omanense’ strains in Iran are quite distinctive, and are mainly represented by witches’ broom and dwarfing in *Convolvulus arvensis*; yellowing, reddening, die-back, and decline in *Prunus persica* and *Prunus domestica*; witches’ broom in *Cressa cretica,* and yellowing and dwarfing in *Sophora alopecuroides* [15,38,39]. They were detected in diverse plant species, and in some case, they also have some molecular differences in their 16S rRNA gene; however, they share geographically close areas in Iran. On the other hand, Middle Eastern countries are the only areas where these phytoplasmas were identified and detected until now. Moreover, comparing the available sequences of strains of ‘*Ca*. P. omanense’, it was possible to verify that the strains detected in these geographic areas cluster in three clades well supported by the phylogenetic analyses. The phytoplasma strains in these clades are geographically substantially separated; it appears that the ‘*Ca*. P. omanense’ strains identified from Iran cluster together, and the same is true for strains from Oman, Jordan and Lebanon, which are all located across the Persian Gulf in front of Iran.

The second detected phytoplasma is a strain of ‘*Ca*. P. australasiae = australasiaticum’, and it is enclosed in the 16SrII-D subgroup. Among the reported phytoplasmas, the 16SrII is the most important and widespread in Iran, identified in the central and southern regions, which have tropical and subtropical climates. This phytoplasma is also reported to infect agronomically relevant crops in Iraq, Oman and Egypt [40,41,42,43,44,45]. It must be taken into consideration that persimmon trees in the orchard of Yazd province are mixed with other pome and stone fruits, in which 16SrII-D phytoplasma strain-associated diseases were reported in other areas of Iran, such as in *Prunus dulcis* with die-back and *Punica granatum* with little leaf [19,46]. ‘*Ca*. P. australasiae = australasiaticum’ strains associated with alfalfa witches’ broom and sesame phyllody were already reported in the areas of the Yazd province where persimmon trees infected with 16SrII-D are detected. The major symptoms of alfalfa witches’ broom phytoplasma (AWB) disease include little leaf, internode shortening, flower virescence and phyllody, and the majority of phytoplasmas identified in alfalfa farms in the country belonged to the 16SrII–D subgroup [13,47]. The major symptoms of sesame infected with these phytoplasmas are phyllody, virescence, little leaf, witches’ broom and shoot proliferation, seed capsule cracking, and seed germinating in the capsule.

In many orchards of fruit trees in Mehriz, alfalfa and sesame are planted in adjacent fields. The insect vector of ‘*Ca*. P. australasiae = australasiaticum’ in both sesame and alfalfa is *Orosius albicinctus* Distant [14,46,47]. According to the fact that phytoplasma strains in the 16SrII-D subgroup were detected in persimmon trees and considering that alfalfa and sesame fields in Yazd province were infected with phytoplasmas of the same ribosomal subgroup, there is a possibility that alfalfa and sesame may contribute to the disease epidemiology by serving as inoculum sources for their transmission by this insect vector to persimmon. This hypothesis is strengthened because the *O. albicinctus* was identified in the orchards with infected trees (S.A. Esmaeilzadeh-Hosseini, unpublished). Therefore, persimmons may play an important role in the epidemiology of ‘*Ca*. P. australasiae = australasiaticum’ diseases in this geographic area where phytoplasma-associated diseases are quite commonly detected in herbaceous host species [48]. The detection by RFLP analyses in some of the samples of profiles of ‘*Ca*. P. solani’ in a mixed infection with the other detected phytoplasmas further suggests the possibility of an epidemiological cycle in which alfalfa play a relevant role, since it was found to be infected by this and 16SrII-D phytoplasmas as well in Iran [14]. The lack of sequencing of the phytoplasma is linked to its mixed infection presence in the tested samples and to the lack of specific primers working appropriately under the mixed infection conditions, as reported above.

Due to the rapid spreading in the last few years of ‘*Ca*. P. omanense’ in Iran, especially in fruit trees, and also due to the presence of a destructive decline that severely damages fruit trees, there is an urgent need for accurate surveys to verify the presence and identity of phytoplasmas in fruit trees. In particular, a wide investigation of the presence of ‘*Ca*. P. omanense’ strains as an emerging threat to fruit orchards should be performed. However, these findings and their implications should be considered in the broadest context possible to devise the most appropriate management features to contain the spreading of both these phytoplasmas, which are infecting a large number of different crops in Iran and in the neighboring geographic areas.

In the orchard where the symptomatic persimmon trees were located, active insects were collected using an insect net and placed in a 50 cm × 50 cm × 50 cm mesh cage, while leafhoppers were collected with an aspirator. Different species of leafhoppers caught in the fruit orchards and their garden grasses were examined for the presence of phytoplasma using nested PCR assays, and the 16SrII phytoplasmas were the only ones detected in *Orosius albicinctus*. However, other species of leafhoppers collected, including *Austroagallia sinuata*, *Anacertagllia laevis*, *Neoaliturus fenestratus*, *N. guttulatus*, and *Psammotetix striatus*, are present in these areas in Iran, and could act as vectors for this phytoplasma [49]. Moreover, in Oman, *Austroagallia avicula* and *Empoasca* sp. collected in symptomatic alfalfa fields were found to be infected with 16SrII-D phytoplasmas [50] and can be considered further potential vectors. Further studies are thus also needed on insect vector identification to devise the most suitable management practices to reduce the spreading and incidence of these phytoplasmas in persimmon and in other agronomically relevant plants, as well as the environmental species that could act as effective alternative host species for these and other phytoplasmas.

## Figures and Tables

**Table 1:** Lineages suggested from restriction sites’ positions of ‘*Ca*. P. omanense’ strains available in GenBank marked with different colors compared with the one of persimmon after virtual RFLP with *Aat*II and *Bcg*I restriction enzymes (in bold, reference strain and persimmon strain).

Host Plant Species	Strain	Country	GenBank Accession Number	*Aat*II	*Bcg*I
*Vitis vinifera*	VV1259	Jordan (A)	OL873117	910	831/281 865/247
***Cassia italica***	**IM-1**	**Oman (A)**	**EF666051**	**910**	**831/281** **865/247**
***Diospyros kaki***	**Mehriz 1**	**Iran**	**PP829290**	**1112**	**796/281** **864/247**
*Prunus persicae*	Ft4	Iran	MF142457	1112	796/281 864/247
*Vitis vinifera*	CAPO-LEB	Lebanon	LN874219	1112	796/281 864/247
*Prunus domestica*	PD1075	Jordan (B)	OR736054	1112	831/281 865/247
*Pyrus communis*	PE704	Jordan (B)	OR295224	1112	831/281 865/247
*Vitis vinifera*	VV95	Jordan (B)	OL873118	1112	831/281 865/247
*Cassia Italica*	IM-4	Oman (B)	EF666054	1112	831/281 865/247
*Convolvolus arvensis*	Conv10	Iran	KY047493	**1113**	**797**/281 865/247
*Sophora alopecuroides*	Sop2	Iran	MN219986	**1113**	**796/282** **864/248**
*Sophora alopecuroides*	Sop1	Iran	MN219988	**1120**	**796/289** **864/255**

## Data Availability

The data presented in this study are openly available in GenBank NCBI under accession numbers PP829290, PP829288, and PP829289.
